# Musculoskeletal Disorders and Psychological and Environmental Factors Associated with Recreational and Sport Fishing: A Narrative Review

**DOI:** 10.3390/jfmk11010018

**Published:** 2025-12-30

**Authors:** Paweł Pędrasik, Bartosz Wilczyński, Katarzyna Zorena

**Affiliations:** Department of Immunobiology and Environmental Microbiology, Faculty of Health Sciences, Medical University of Gdańsk, 80-210 Gdańsk, Poland; bartosz.wilczynski@gumed.edu.pl

**Keywords:** angling-related health, outdoor physical activity, green-space therapy, overuse injuries, acute injuries, psychosocial well-being, nature exposure, therapeutic outdoor activities

## Abstract

Fishing is a widely practiced recreational activity that offers psychological, physical, and social benefits, but it also poses risks such as acute trauma and chronic overuse injuries. This narrative review aims to (1) synthesize current evidence on the musculoskeletal disorders, psychological outcomes, and environmental factors associated with recreational and sport fishing; (2) identify the physical, mental, and social health benefits reported across different angling disciplines; (3) characterize acute and chronic injury risks, including overuse syndromes and environment-related hazards; and (4) highlight gaps in the literature to guide future research directions in public health, rehabilitation, and preventive medicine. Materials and Methods: A narrative review was conducted in accordance with SANRA guidelines. A structured search of PubMed, Scopus, Web of Science and Google Scholar identified studies published between 2000 and 2025. Eligible sources included population surveys, clinical studies, therapeutic angling programs, epidemiological reports, and case studies addressing physical, psychological, or injury-related outcomes in recreational or sport fishing. Studies on commercial or occupational fishing were excluded. Evidence was synthesized thematically across benefit and risk domains. A total of 565 records were identified across four databases (PubMed, Scopus, Web of Science, Google Scholar). After screening, duplication, and full-text assessment, 41 studies met the eligibility criteria and were included in the narrative synthesis. The evidence indicates significant psychological benefits of fishing, including reductions in stress, improved mood, and clinically meaningful decreases in Post-Traumatic Stress Disorder (PTSD) symptoms reported in therapeutic fly-fishing programs. Musculoskeletal outcomes were more heterogeneous: chronic conditions such as low back pain and repetitive strain injuries of the shoulder, elbow, and wrist were commonly reported among regular anglers, particularly in physically demanding disciplines. Ice and sea fishing were associated with distinct environmental risks, including hypothermia, frostbite, and rare but documented fatal incidents. The results of this narrative review highlight the therapeutic potential of both recreational and sport fishing. However, they also point to the need for greater awareness of the risk of injury and environmental hazards associated with this type of fishing.

## 1. Introduction

Fishing is one of the world’s most widely practiced outdoor activities, combining recreation, physical activity, and a range of techniques from casual angling to competitive sport fishing [1]. While other sports such as running, cycling, and team sports have been extensively studied for their health benefits, risks, and injury profiles, fishing has not been systematically evaluated to the same extent despite its global popularity and cultural significance [2,3,4]. Understanding its therapeutic potential and injury risks is important for public health, rehabilitation, and preventive care.

Fishing is both a leisure activity and a professional sport with deep cultural roots and significant economic importance. In developed countries, participation ranges from 2% to over 11% of the population [5,6,7]. In the United States, recreational fishing represents one of the largest outdoor industries and accounts for a substantial share of economic activity in certain states, generating approximately USD 125 billion in annual economic impact nationwide. According to the American Sport fishing Association, fishing is a hobby pursued by some 40 million Americans [1,8]. In Australia, recreational fishing generates higher revenues than commercial fisheries in some regions [9]. In Poland, an estimated 1.5 to 2 million people actively fish, including approximately 600,000 members of the Polish Angling Association (PZW) [10].

Fly fishing, with a rich history dating back to The Compleat Angler (1653), is deeply embedded in the cultural identity of countries such as the United Kingdom, New Zealand, and the United States, where 6.5 million people practiced it in 2016 [11]. Although research has primarily focused on fly fishing injuries—such as overuse conditions and back pain—other fishing disciplines (spinning, marine, feeder, float) remain underexplored [12,13].

Although participation, economic factors, environmental conditions, and injury mechanisms are introduced as context, they directly inform the physical, psychological, and social health pathways examined in this review. These connections provide the rationale for the research questions guiding the study.

Growing evidence indicates that recreational and sport fishing may provide meaningful health benefits across psychological, physical, and social domains. Regular angling participation has been associated with stress reduction, improvements in mood, enhanced attentional control, and reduced symptoms of depression, anxiety, PTSD, and ADHD [14,15,16,17,18]. Physically, fishing involves light-to-moderate activity that can support balance, coordination, and functional fitness, particularly in older adults. Socially, angling fosters community engagement, intergenerational bonding, and structured outdoor routines that contribute to overall well-being [6,15,19].

The potential therapeutic effects of fishing are supported by several physiological and neurobiological mechanisms. Exposure to natural environments activates parasympathetic responses, lowers cortisol levels, and aligns with established models of “green exercise,” including the Stress Recovery Theory and Attention Restoration Theory [18,20,21]. Repetitive, rhythmic casting movements may enhance motor control and encourage mindfulness-like cognitive states. Additionally, the anticipatory reward processes inherent in angling—such as focus, delayed gratification, and goal-directed behavior—engage dopaminergic pathways linked to motivation and emotional regulation. Dopamine neurons encode discrepancies between expected and received rewards, and respond strongly to uncertain or delayed reinforcement, providing motivational drive for goal-directed behavior. Qualitative accounts of therapeutic fly-fishing describe states of focused attention, reduced rumination and present-moment awareness, overlapping conceptually with mindfulness constructs [6,20,21,22,23,24,25,26,27]. Yet these models account only partially for the heterogeneity of outcomes observed in angling, indicating areas where established theories do not fully apply. Moreover, recent evidence suggests that recreational angling participation is also sensitive to broader environmental and societal conditions. A longitudinal study from coastal Louisiana (2015–2021) demonstrated that weather variables, particularly temperature, were key drivers of fishing effort, with hurricanes and tropical storms leading to short-term declines followed by rapid recovery [28]. Notably, during the COVID-19 pandemic, fishing effort more than doubled in some months compared to pre-pandemic levels, emphasizing the role of angling as an adaptive and resilient form of outdoor recreation under global stressors [28].

Despite these emerging insights, the current evidence base remains fragmented. Most available studies focus on fly fishing, therapeutic angling programs, or narrow clinical populations. There is a lack of large-scale epidemiological studies, biomechanical analyses, randomized controlled trials, and research covering diverse fishing disciplines such as spinning, feeder, carp, sea, and ice fishing. Data from Central and Eastern Europe, low- and middle-income countries, and non-Western contexts are particularly limited. Furthermore, chronic musculoskeletal risks, acute injuries, long-term psychological outcomes, and environmental exposures remain under-investigated, underscoring the need for a consolidated synthesis.

## 2. Objectives

This narrative review aims to (1) synthesize current evidence on the musculoskeletal disorders, psychological outcomes, and environmental factors associated with recreational and sport fishing; (2) identify the physical, mental, and social health benefits reported across different angling disciplines; (3) characterize acute and chronic injury risks, including overuse syndromes and environment-related hazards; and (4) highlight gaps in the literature to guide future research directions in public health, rehabilitation, and preventive medicine.

## 3. Materials and Methods

This narrative review was conducted in accordance with the SANRA (Scale for the Assessment of Narrative Review Articles) checklist, to ensure methodological transparency and rigor [29]. The decision to conduct a narrative review was driven by the substantial heterogeneity of the available evidence. Studies on recreational and sport fishing vary widely in terms of population characteristics, study designs, outcome measures, fishing techniques, and health domains assessed (mental health, musculoskeletal load, physical activity, environmental risks, and social functioning). This diversity precludes the use of standardized methodological approaches required for systematic reviews or meta-analyses, which rely on narrowly defined research questions, homogeneous outcomes, and comparable methodological quality across studies. A narrative synthesis was therefore the most appropriate approach to integrate interdisciplinary findings, identify overarching patterns, and map conceptual relationships across diverse strands of evidence. This method also aligns with the objectives of the present study, which aimed to provide a broad, integrative overview of health-related factors associated with recreational and sport fishing.

### 3.1. Databases and Search Strategy

A structured literature search was conducted in PubMed, Scopus, Web of Science, and Google Scholar for articles published between January 2000 and June 2025. This period was selected to reflect modern angling techniques, equipment, therapeutic programs and current musculoskeletal and psychological research relevant to fishing. Language restrictions (English and Polish) were applied to ensure accurate screening and consistent interpretation of psychological, clinical, and biomechanical terminology.

### 3.2. Database-Specific Search Strategies

Search strategies were adapted to the structure and indexing rules of each database: PubMed: Combined MeSH and free-text terms using the following Boolean string: (“recreational fishing” [tiab] OR “sport fishing” [tiab] OR angling [tiab] OR “fly fishing” [tiab]) AND (“musculoskeletal injuries” [tiab] OR “overuse injury” [tiab] OR tendinopathy [tiab] OR “shoulder pain” [tiab] OR epicondylitis [tiab] OR “upper extremity pain” [tiab] OR “low back pain” [tiab] OR “mental health” [tiab] OR psychological [tiab] OR stress [tiab] OR “PTSD” [tiab] OR “well-being” [tiab] OR “outdoor therapy” [tiab] OR “therapeutic recreation” [tiab]) NOT (aquaculture [tiab] OR “commercial fishing” [tiab] OR fisheries [tiab] OR shark [tiab] OR whale [tiab] OR “bycatch” [tiab] OR “marine ecology” [tiab] OR “fish behavior” [tiab] OR “climate change” [tiab] OR “marine” [tiab] OR “occupational fishing” [tiab]).

- **Scopus:** Searched using TITLE-ABS-KEY fields:

TITLE-ABS-KEY (fishing OR angling OR “recreational fishing” OR “sport fishing”) ANDTITLE-ABS-KEY (“fly fishing” OR spinning OR “lure fishing” OR “float fishing” OR “feeder fishing” OR “carp fishing” OR “ice fishing” OR “sea fishing” OR “boat fishing”) ANDTITLE-ABS-KEY (injury OR injuries OR trauma OR “overuse injury” OR musculoskeletal OR “chronic pain” OR “mental health” OR psychological OR wellbeing).

- **Web of Science:** Searches performed using the TOPIC field:

TS = ((“recreational fishing” OR “sport fishing” OR “fly fishing” OR “ice fishing”)AND (“hook injury” OR “ocular injury” OR “fish hook” OR “musculoskeletal disorder” OR “overuse injury” OR “upper extremity pain” OR “shoulder pain” OR “elbow pain” OR “back pain” OR “post-traumatic stress” OR “PTSD” OR “mental health” OR “psychological wellbeing”) AND (Human *) NOT (commercial OR industry OR occupational OR aquaculture OR fishery OR fisheries)).

- **Google Scholar:** Simplified search due to limited Boolean functionality:

“recreational fishing” (injury OR trauma OR “musculoskeletal” OR “mental health”) -ecology -fishery -commercial -aquaculture.

### 3.3. Eligibility Criteria

**Inclusion criteria:**

- Studies involving human participants;
- Articles published in English or Polish;
- Publications addressing recreational or competitive fishing and its physical, psychological, or social health effects;
- All study designs, including observational studies, clinical reports, surveys, therapeutic program evaluations, and case reports.

**Exclusion criteria:**

- Studies focusing solely on commercial or occupational fishing injuries;
- Non-original content such as editorials, commentaries, or popular science articles without empirical data;
- Publications without health-related outcomes.

### 3.4. Additional Sources

In addition to peer-reviewed studies, this review included publicly available reports from health authorities, governmental agencies, and professional organizations (e.g., WHO, CDC, NHS, CIPS, PZW). Non-peer-reviewed sources were considered if they provided relevant epidemiological or population-level data on angling participation, injury rates, or health outcomes.

### 3.5. Study Selection and Screening Process

Two independent reviewers screened all titles and abstracts for relevance. Full texts were assessed against inclusion criteria. Any discrepancies were resolved through discussion or consultation with a third reviewer.

### 3.6. Quality Appraisal

The methodological quality of this narrative review was assessed using the SANRA (Scale for the Assessment of Narrative Review Articles) checklist. The review met all SANRA criteria. The importance of the article was strongly justified based on the clear gap in current knowledge regarding fishing and health (score 2). The aims and research questions were well defined and explicitly formulated (score 2). The literature search strategy was detailed, involved multiple databases, and followed transparent procedures (score 2). Referencing was complete, current, and formatted according to standardized Vancouver guidelines (score 2). Scientific reasoning was coherent and evidence based, demonstrating clear synthesis across studies (score 2). Finally, the presentation of data was appropriate, incorporating structured summaries, tables, and figures to support clarity and readability (score 2).

### 3.7. Synthesis of Evidence

Extracted data were thematically grouped into two primary domains:

1. Benefits—mental health, physical activity, and social integration.
2. Risks—acute injuries, overuse injuries, environmental factors, and rare accidents.

### 3.8. Bias and Limitations

Potential biases related to study selection (publication bias, language bias) and limitations inherent to narrative synthesis are acknowledged and discussed in the Discussion section.

## 4. Results

A total of 565 records were identified across four databases (PubMed, Scopus, Web of Science, Google Scholar). After title and abstract screening, 524 articles were excluded. Following full-text assessment and deduplication, 41 studies met the eligibility criteria and were included in the narrative synthesis.

From PubMed, 30 records were retrieved; 20 were excluded after screening, leaving 10 studies for inclusion. The Scopus search yielded 51 records; 28 were excluded based on title and abstract, 7 were identified as duplicates, and 16 unique studies were retained. From the Web of Science Core Collection, 14 records were identified; 8 were excluded, 4 were duplicates of studies already captured in PubMed or Scopus, and 2 unique studies were included. The Google Scholar search produced 470 records; 14 met the inclusion criteria on initial screening, of which 1 was a duplicate, resulting in 13 unique studies.

The included studies covered a diverse set of research domains, encompassing psychological and behavioral outcomes, environmental influences, biomechanical and musculoskeletal aspects, epidemiological patterns (including case reports), therapeutic or rehabilitative programs, as well as cross-sectional and longitudinal population-level observations.

Across the included studies, psychological outcomes were generally consistent, whereas musculoskeletal findings showed greater variability across angling disciplines. Differences in study design and measurement tools contributed to heterogeneity. Overall, the evidence is moderate for psychological effects, limited for musculoskeletal outcomes outside fly fishing, and preliminary for social impacts.

**Figure 1:**
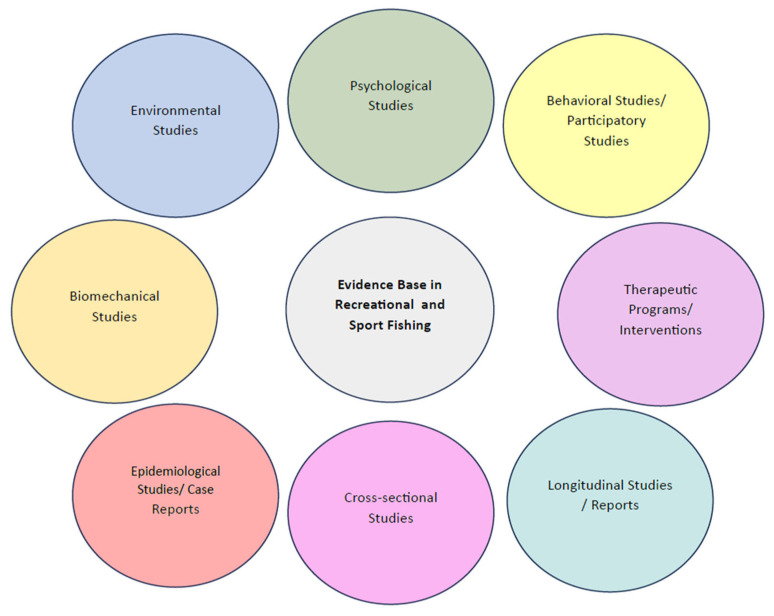
Conceptual map of the evidence base in recreational and sport fishing.

To illustrate the structure of this evidence base, the main domains identified in the literature are summarized in Figure 1.

### 4.1. Disciplines and Techniques in Angling

Angling encompasses a wide variety of disciplines, which differ in terms of fishing technique, equipment used, and fishing environment. The diversity within the sport is extensive. Fishing disciplines refer to broad categories of angling that vary depending on purpose (e.g., spinning, float fishing, fly fishing) and environment (e.g., marine, inland or ice fishing) [30]. Fishing methods or techniques, on the other hand, are more specific terms that describe the particular approaches used within each discipline. Among the recognized disciplines are: casting (precision casting), float fishing, feeder fishing, carp fishing, fly fishing, spinning, ice fishing, and surfcasting. There are also specialized forms such as float fishing for individuals with physical disabilities [30,31].

More demanding types of angling, such as salmonid fishing in rivers or at sea using fly or spinning methods, require traveling long distances through difficult terrain, wading in water, and enduring strong currents and adverse weather conditions. Other active angling disciplines—such as shore or boat spinning—are physically less intense. These gradually give way to stationary methods such as float fishing, feeder fishing, or carp angling [10,30,31,32,33,34].

### 4.2. Fishing and Human Health

Numerous studies have confirmed the therapeutic impact of fishing on mental health [11,17,23,35,36,37,38,39,40]. The psychological benefits are further supported by large-scale observational data from the United States. A long-term observational dataset analysis of recreational fishing in coastal Louisiana demonstrated that, during the first year of the COVID-19 pandemic, angling effort more than doubled in some months compared to pre-pandemic levels [28]. This surge in participation highlights the role of recreational fishing as a safe, accessible, and resilient coping strategy during global crises, reinforcing its value in stress reduction and mental health support [24,41,42]. One such study—Mental Health and Recreational Angling in UK Adult Males: A Cross-Sectional Study—conducted on a sample of 1752 participants, investigated the relationship between angling status (defined by the frequency, regularity, and duration of fishing activity) and the prevalence of mental health conditions such as depression, schizophrenia, suicidal ideation, and deliberate self-harm. The study revealed that individuals who engaged in fishing regularly experienced better mental well-being and reported fewer symptoms of depression and anxiety compared to those who fished less frequently. These associations were determined using age-adjusted regression models, which showed that more frequent angling was associated with lower odds of developing mental health disorders. The authors reported a statistically highly significant reduction in the risk of depression, schizophrenia, and suicidal ideation (*p* < 0.001), as well as a statistically significant decrease in the risk of deliberate self-harm (*p* < 0.012) among regular anglers [19].

### 4.3. Fishing as a Form of Psychotherapy

Promising research findings have led to growing interest in angling as a therapeutic intervention. In England, the National Health Service (NHS) has partnered with the charitable organization Tackling Minds to promote angling for individuals struggling with anxiety and depression [43].

Furthermore, this therapeutic potential is being explored through more targeted studies and initiatives, such as the development of secondary school curricula that promote positive mental health by fostering connection and a sense of meaning. One such example is the Canadian initiative titled “Fishing for Meaning: Developing a Nature-Based Therapy Program with a Fly-Fishing Guide for Students at the Education Support Centre of Prince George Secondary School.”

Based on the evolving role of schools in addressing mental health challenges, the government of British Columbia—in collaboration with the Canadian School Health Consortium—developed a bonding strategy focused on strengthening relationships, maintaining positive emotions, and increasing resilience. In 2018, the British Columbia government emphasized school connectedness as the key factor influencing youth mental health.

Within this framework, schools are encouraged to implement programs that promote positive emotions, engagement, relationships, meaning, and accomplishment—commonly referred to by the acronym PERMA. The project aims to create a course that enables the most vulnerable students at Prince George Secondary School (PGSS) to pursue the goals outlined in the PERMA strategy, in accordance with British Columbia’s new curriculum. To achieve this, a Nature-Based Therapy approach will be employed, combining fly fishing-related content with experiential learning aligned with key competency development [44].

In addition to targeted therapeutic interventions and school-based initiatives like Fishing for Meaning, the UK has also pioneered practical models linking angling to education. One notable example is the “Fishing for Schools” program, launched in 2007. Initially designed to support students with learning difficulties, the program used angling as a medium to re-engage pupils with formal education and build self-confidence through experiential outdoor learning.

By 2012, interest from mainstream schools had grown significantly, recognizing the value of angling-based instruction for a broader range of students. Today, Fishing for Schools offers a unique and inclusive educational provision for young people from diverse backgrounds and ability levels. The initiative complements broader efforts to harness nature-based activities—such as fishing—not only as a therapeutic tool, but also as a pathway to personal development, skill-building, and academic engagement [45].

Another notable example of angling as a tool for social development is the UK-based charity Get Hooked on Fishing. This organization works to create brighter futures for young people by offering fun, structured and interactive experiences around the sport of angling. Designed in collaboration with youth participants, the program aim to build confidence, encourage positive behavioral change, and show that alternative and constructive life pathways are possible. By engaging young people through sport angling activities, the initiative contributes not only to their personal development but also to stronger, safer communities [46,47,48].

Although programmers such as Fishing for Meaning, Fishing for Schools, and Get Hooked on Fishing demonstrate how angling can be used in educational, therapeutic, and community settings, most available evidence describing their impact is qualitative. The Social & Community Benefits of Angling project, for example, documents improvements in confidence, social skills, wellbeing, and community engagement, yet these findings derive from interviews, observations, and descriptive case studies rather than controlled or quantitative evaluations. These initiatives therefore illustrate the practical potential of angling-based interventions but do not yet provide robust empirical data to confirm their effectiveness [40,43,45,46].

A 2019 study conducted among 244 recreational sea anglers in Spain revealed significant relationships between angling activity, stress levels, seafood consumption, and sleep quality. The average stress index among anglers was moderate, at 36.4 units. Fishing activity showed a positive effect on stress reduction, with each additional hour of monthly fishing associated with a 0.016-unit decrease in stress levels. Anglers who spent more time fishing experienced 15.4% lower stress levels compared to less active anglers [49].

The stress index used in the study (ranging from 14 to 70) demonstrated high internal consistency (α = 0.83), indicating the reliability of the tool. Anglers reported good sleep quality, with a low sleep problem index of 39.5 units. These findings suggest that fishing may have a positive impact on sleep quality and overall well-being [49].

Lower scores on the negative affect scale further indicated that anglers experienced fewer negative emotions such as fear, anger, or nervousness. Regular angling activity supports a sense of calm and emotional balance. As an outdoor activity, fishing provides contact with the natural environment, which is beneficial to mental health. Spending time in nature reduces psychological tension and can support stress management and emotional regulation [41,50,51,52].

Although empirical research on fishing and ADHD is limited, anecdotal evidence suggest potential benefits, such as improvements in patience, focus, and self-regulation [16].

Fishing may therefore serve as a therapeutic tool for individuals facing mental health challenges, helping to improve mood, reduce tension, and support mental health in a holistic manner [18,49,53].

In recent years, a growing body of research has supported the use of movement-based therapeutic interventions for individuals who have experienced trauma. Fly fishing is one such example that has been employed in the United States for many years to help war veterans adjust to post-combat life and process traumatic memories [14,15,18,23,24,54].

One notable initiative is Project Healing Waters Fly Fishing (PHWFF), founded in 2005 and initially implemented by the Walter Reed Army Medical Center [55]. This program, which promotes recreational therapy through fly fishing, has since expanded and is now offered at military hospitals and veterans’ clinics across the United States.

This gap highlights the need for more systematic studies exploring fishing as a psychotherapeutic intervention, particularly among youth populations [55,56].

Not all studies report positive psychological or social effects of angling. Some analyses describe neutral or inconsistent outcomes, and many findings come from cross-sectional designs without control groups, making it difficult to separate true effects from selection bias or confounding.

### 4.4. Fishing as Physical Activity and Conditioning

Effective fly casting requires precise coordination, rhythmic timing, and consistent practice. While the basic cast can be learned relatively quickly, mastering the technique can take a lifetime. After the cast is made, the line must be retrieved in a specific manner to properly present the fly. In many cases, retrieving the fly involves counting, rhythm, and tempo, as the angler seeks to imitate the swimming behavior of certain aquatic organisms [57].

One of the most unique aspects of fly fishing is that these rhythmic and structured movements occur in natural surroundings. For school-aged youth, post-mastectomy patients, individuals with cardiac conditions, psychiatric patients, older adults, or veterans, fly fishing offers a purposeful and physically engaging task that supports and enhances both physical and mental well-being. Moreover, it contributes to an improved quality of life and serves as a stimulus for exploring deeper meanings associated with time spent in nature [15,23].

Through this process, participants become more aware of the functioning of diverse aquatic ecosystems and, at the same time, can connect this awareness directly to their own bodily experience. The body becomes not only an instrument of casting and movement, but also a perceptive tool that synchronizes with the flow of water and the rhythm of the natural world [18,21,56].

This therapeutic dimension is not exclusive to fly fishing. Similar benefits may be observed in other forms of angling, particularly those that require bodily engagement, concentration, and attentiveness to environmental cues. Techniques such as spinning, float fishing, or feeder fishing also demand coordination, controlled motor patterns, and postural stability—particularly when practiced in outdoor, often unpredictable, environments [19,49,58].

Although the physical requirements may vary across disciplines, each presents opportunities to promote mindfulness, reinforce functional movement patterns, and encourage outdoor physical activity. As such, angling—regardless of technique—can serve as an accessible and meaningful form of therapeutic physical engagement, especially for individuals recovering from illness, managing chronic health conditions, or seeking psychosocial rehabilitation through structured nature-based interventions [59,60].

According to current research, fishing significantly contributes to physical activity and impacts the musculoskeletal system of those who participate in it. In relevant studies, anglers reported engaging in moderate to vigorous physical activity for approximately 2.5 h per day. Part of this activity likely occurs during fishing itself, where the estimated metabolic equivalent of task (MET) values are as follows: 3.5 METs for general fishing, 3.5 METs for riverbank fishing, and 6.0 METs for wading in streams or rivers [61]. Fishing can meaningfully contribute to physical activity levels, with general fishing ≈ 3.5 METs and wade fishing ≈ 6 METs. Wade fishing, in particular, demands greater physical effort and cardiovascular engagement [61].

A British study reported that a similar proportion of float anglers, sea anglers, and fly fishers (approximately 20%) perceived their participation as a low-intensity physical activity. Most anglers considered their fishing participation to be of moderate intensity, although this perception was less frequent among fly fishers compared to float and sea anglers. Notably, a much higher percentage of fly anglers (34.2% of respondents) classified their fishing activity as high-intensity physical activity.

These findings confirm that wade fishing in rivers is the most physically demanding form of angling, contributing significantly to cardiovascular fitness and physical endurance. However, it also carries a greater risk of injury due to its demanding nature [38].

A study conducted in Galicia, Spain, also highlighted the role of fishing as an important component of physical activity. The intensity of fishing, measured in hours per month, was found to influence participants’ physical health. In addition, other forms of physical activity, such as sports and occupational work, were evaluated and shown to contribute to the overall activity level. The physical activity assessment tool used in the study demonstrated acceptable reliability (α = 0.63), and including these variables allowed for a more comprehensive understanding of the impact of fishing on physical health [49].

#### 4.4.1. Physical Conditioning and Sport-Specific Demands

Recent literature has begun to conceptualize angling—particularly sportfishing—as a structured physical activity that encompasses multiple elements of fitness and motor control. A study by Pratama and Sarkity (2023), analyzing sportfishing within maritime communities, identifies five core physical components involved in this activity: muscular strength, balance, reaction time, explosive power, and endurance [59].

#### 4.4.2. Muscular Strength

Angling, especially when targeting large species, requires considerable upper limb strength, particularly in the arms and shoulders. The ability to cast heavy gear, resist strong currents, and retrieve large fish places significant mechanical demand on the musculoskeletal system. Without sufficient muscular capacity, anglers are at risk of fatigue, cramping, or acute musculoskeletal injuries [59].

#### 4.4.3. Balance

Maintaining static and dynamic balance is crucial for safe and effective fishing, particularly in environments with unstable or slippery terrain (e.g., riverbanks, rocky shorelines, or boat decks). Balance relies on the integration of visual, vestibular, and proprioceptive systems, and is especially critical when wading in streams or casting from irregular platforms. Falls during fishing—often due to impaired balance—are a documented cause of injury and even fatality in some regions (e.g., Brazil) [59].

#### 4.4.4. Reaction Time

The rapid detection of a fish strike and timely initiation of hook-setting require well-developed reaction speed. Anglers must interpret subtle sensory cues (e.g., line movement or tension) and translate them into coordinated motor actions, often within fractions of a second. Deficits in reaction time may result in missed catches or mishandling of equipment [59].

#### 4.4.5. Explosive Power

Beyond static strength, angling necessitates explosive muscular actions, such as quick rod lifts or fast retrievals, especially during aggressive strikes or when handling strong fish. This aspect requires the combined development of strength and speed—collectively referred to as power—and is central to effective performance in sportfishing [59].

#### 4.4.6. Endurance

Angling is often practiced for extended periods, requiring cardiorespiratory and muscular endurance. Long durations of standing, casting, retrieving, and walking—often while carrying gear—demand sustained aerobic capacity and localized muscle fatigue resistance. Low endurance may reduce efficiency, increase injury risk, and negatively impact overall experience and outcomes [59].

These findings emphasize that angling—particularly in its sport-oriented forms—should not be viewed as a passive activity. Rather, it presents clear physical training demands, which can be targeted through structured exercise programs. Incorporating strength, balance, reaction, and endurance training into preparation routines may enhance performance and reduce injury risk among recreational and competitive anglers [59].

### 4.5. Musculoskeletal Load and Overuse Injuries

Although fishing offers numerous health and recreational benefits, it is not free from the risk of injuries and disorders resulting from repetitive movement patterns specific to different angling techniques. Each fishing discipline is associated with unique biomechanical demands placed on the musculoskeletal system, which may lead to acute and chronic pain or injury [57].

Techniques such as fly fishing and spinning often require anglers to wade through rivers over slippery rocks and uneven streambeds in strong currents. When practiced at sea, these methods demand maintaining balance on stony seabeds under challenging environmental conditions—strong winds, tides, and waves. These factors contribute to fishing-related disorders and injuries, which are often a direct consequence of such conditions [62,63].

Equipment and casting technique also play a significant role in injury development. These active fishing methods involve hundreds of casts per day, often using heavy gear, longer and stiffer rods necessary for long-distance casting and fighting large fish. In addition, anglers frequently cover long distances—often carrying substantial gear—which further increases the risk of strain and injury to the musculoskeletal system [57,62,64]. Repetitive overhead arm movements and intensive engagement of the shoulder joint can lead to overuse syndromes, such as rotator cuff tendinitis. Notably, the median number of casts per day among fly fishers in the study by Kuhn and Kuhn was reported to be 200 casts [57,65,66].

The analysis of orthopedic conditions among fly fishers demonstrates patterns similar to those observed in athletes practicing other repetitive sports [67,68,69,70]. Survey data show that 64% of 131 respondents had consulted an orthopedic surgeon, chiropractor, or general practitioner for fishing-related musculoskeletal complaints. Additionally, 35% had undergone orthopedic surgery, 36% had received physical therapy, 16% had received manual therapy, and 40% had been treated with prescription pain medications due to fly fishing-related conditions [57]. Catfish and saltwater angling typically require the use of particularly heavy gear—including large rods, reels, and baits—and may involve physically demanding encounters with trophy-sized fish weighing several dozen kilograms, and in the case of large species such as tuna or sturgeon, even hundreds of kilograms [71].

It should also be noted that the current evidence base is heavily skewed toward fly fishing, which is reflected in the predominance of upper-limb and musculoskeletal studies devoted to this discipline. Although fly fishing represents only a small fraction of global angling participation, it is the focus of most available biomechanical, orthopedic, and overuse-injury research [13,24,57]. In contrast, quantitative data on spinning, float fishing, feeder fishing, carp angling, marine fishing, and ice fishing remain extremely limited and are often restricted to isolated reports or emergency-department datasets [72,73]. Consequently, findings derived from fly-fishing populations should not be generalized to all angling techniques; rather, they represent the best-documented segment of the evidence, not the full spectrum of fishing practices.

Given this imbalance in the evidence base, it is important to emphasize that several commonly cited findings—particularly those related to musculoskeletal load, upper-extremity overuse patterns, and biomechanical demands—originate almost exclusively from fly-fishing studies [57]. Although these data offer valuable insight into potential injury mechanisms, they cannot be assumed to apply uniformly across all angling disciplines. Techniques such as spinning, float, feeder, carp, marine, and ice fishing involve distinct movement patterns, environmental exposures, and equipment characteristics, which may lead to different profiles of health benefits and risks. Therefore, the general statements presented in this review should be interpreted as summarizing the best-documented portion of the literature rather than as universal characteristics of all forms of recreational and sport fishing.

#### 4.5.1. Environmental Factors Contributing to Musculoskeletal Disorders (MSDs)

While fishing is generally viewed as a safe outdoor pastime, it can still lead to chronic musculoskeletal strain and, less commonly, acute traumatic injuries. Effective prevention strategies include:

- Mastering proper casting technique;
- Maintaining physical conditioning, particularly of stabilizing muscle groups;
- Conducting preseason and in-season physical preparation;
- Using appropriate, well-balanced gear to minimize strain and the risk of injury.

Regular medical check-ups, early rehabilitation interventions, and injury-specific education should be encouraged for individuals who fish frequently, especially in physically demanding environments [74,75].

To ensure clarity regarding the scope of the evidence, we note that the studies discussed in this subsection include findings derived from three types of sources: (1) research conducted directly on anglers, (2) occupational and general-population studies examining exposure to cold, load, or environmental strain, and (3) mixed or indirect evidence relevant to mechanisms of musculoskeletal load. The non-angling studies are not presented as direct evidence about fishing; rather, they are included to provide mechanistic context and to explain how similar environmental conditions—such as cold exposure, unstable terrain, or repetitive upper-limb activity—may influence musculoskeletal risk during recreational and sport fishing. This clarification addresses the heterogeneity of the available evidence and delineates fishing-specific findings from contextual physiological and ergonomic data.

Environmental conditions may significantly exacerbate the risk of injury. Fishing is often conducted in cold and wet environments, which can negatively affect muscle function, joint flexibility, and recovery. Prolonged exposure to low temperatures reduces blood circulation, increases muscle stiffness, and raises the likelihood of overuse injuries and strain-related conditions [73,76].

Multiple studies have confirmed that long-term cold exposure is associated with higher incidences of musculoskeletal pain, including hand and shoulder disorders, neck and back pain, and carpal tunnel syndrome [77]. Anglers—especially those who fish in rivers, streams, or coastal environments—may be particularly vulnerable due to extended contact with cold water and wind, which intensify physical fatigue and decrease proprioceptive function [33,78,79].

For example, a study from northern Sweden found a significant association between occupational cold exposure and pain in the hands and upper arms. Individuals exposed to cold temperatures for more than half of their working time had notably higher odds of experiencing hand pain (OR: 2.30; 95% CI: 1.23–4.29) and upper arm pain (OR: 1.57; 95% CI: 1.00–2.47) compared to those with less exposure [80].

Another prospective population-based study, also from northern Sweden, revealed that high occupational cold exposure was linked to a greater frequency of neck and shoulder pain (OR: 1.50), low back pain (OR: 1.61), and radiating low back pain (OR: 1.87). These associations remained statistically significant even after controlling for confounders such as age, sex, BMI, smoking status, psychological stress, and physical workload [81].

Research on cold-related hand exposure showed strong links to carpal tunnel syndrome (CTS). Workers reporting frequent hand cooling had significantly higher odds of CTS symptoms (OR: 3.20), and those exposed to cold ambient environments had even higher odds (OR: 4.02) [77].

Longitudinal data from The Tromsø Study in Norway, spanning 7–8 years, indicated that individuals working in cold environments ≥ 25% of their working time had an increased risk of developing musculoskeletal complaints (IRR: 1.15; 95% CI: 1.03–1.29), although the study did not find a statistically significant relationship with severe or multi-site disorders [76]. Environmental conditions also play a critical role in shaping participation and health risks associated with recreational angling. Large-scale observational data from coastal Louisiana demonstrated that weather variables, particularly temperature, were consistent drivers of fishing effort, while tropical storms and hurricanes temporarily reduced participation before a rapid rebound occurred [28]. Such abrupt fluctuations in activity patterns may predispose anglers to musculoskeletal strain after periods of inactivity, highlighting the dual role of environmental conditions both as direct physical stressors and as modifiers of angling behavior [28].

These findings emphasize the need for preventive strategies, including proper warm-up, ergonomic equipment selection, layered thermal clothing, and breaks to reduce cold-induced strain. Awareness and education on these risks are essential to preserving anglers’ physical well-being over time [82].

#### 4.5.2. Upper Limb Injuries

Fly fishing is a technically demanding method that requires rhythmic and coordinated movements for effective casting. Research has shown that the biomechanical demands of fly casting can lead to pain or injury in the shoulders, elbows, and wrists in some individuals [65,83]. Notably, differences in casting styles, techniques, and equipment used may predispose anglers to specific types of injuries [57].

In 2001, Berend conducted an online survey of 89 fly fishers and interviewed an additional 42 members of a local angling club. He found that anglers fishing in saltwater environments most commonly reported shoulder and elbow pain, whereas those targeting trout in inland freshwater systems most frequently experienced wrist pain. Shoulder pain, referred to as “Caster’s shoulder,” was reported by 24% of respondents and was more prevalent among saltwater anglers (31%). This is likely related to the use of heavier gear and long-distance casting, both of which can overload the rotator cuff and contribute to shoulder instability. This prevalence is lower than in other overhead sports such as volleyball or swimming (44%), likely due to differences in casting distances and fish size between freshwater and saltwater ecosystems, which directly influence the physical strain of retrieving a catch [57,84].

Another common condition reported was “Flyfisher’s elbow,” resembling lateral epicondylitis (tennis elbow) or medial epicondylitis (golfer’s elbow). It was most frequently observed among saltwater fly fishers (30%) who typically use heavier rods and longer casts with a double-haul technique. In contrast, warmwater anglers reported the lowest incidence of elbow pain (12%).

Wrist and hand pain was the second most commonly reported issue (26% of respondents). Paradoxically, it was least frequent among saltwater anglers (12%) and most common among freshwater anglers (31%), where precision casting and finer wrist motions are required. The injury mechanism may resemble conditions such as De Quervain’s tenosynovitis or intersection syndrome, both of which are common in racquet sports [57].

In the United States, several preliminary studies have focused on fly fishing due to the high participation rate in this discipline. In December 2022, a dedicated survey was conducted among fly anglers recruited from major online fly-fishing forums. The study included 254 respondents, the majority of whom were men over the age of 65 (69.41%) and right-handed (88.24%). Most were retired (57.09%) and had an average of 34.07 years of fly-fishing experience, fishing an average of 53.37 days per year [64].

More than half of participants (51.39%) reported experiencing upper limb pain or discomfort, most commonly in the shoulder (30.58%). In most cases (92.86%), the pain resolved within minutes to hours after fishing, while only a small portion experienced chronic symptoms (6.35%). Nearly half (48.82%) reported some level of difficulty in fly fishing due to upper limb pain, and among those who reported pain, 44.53% (22.44% of all respondents) sought medical attention. Physical therapy was rarely reported (1.18%), though approximately 20.87% had undergone orthopedic surgery for upper limb conditions [83].

In another study of 162 fly fishers, 59 individuals (36.4%) reported upper limb pain or soreness immediately after fishing. The most frequently affected area was the shoulder (44.7%), followed by the elbow (16.0%) and the hand (16.0%). Pain occurred more often on the right side (79.8%) and was rated at 4.0 (IQR 3.0–6.0) on a 10-point Likert scale. It typically lasted less than one day (45.0%) or from one day to one week (45.0%). Most respondents (62.7%) did not require medical consultation. Among those who did, the most common diagnoses included elbow tendinitis (6 cases), rotator cuff tendinitis (6 cases), and carpal tunnel syndrome (5 cases) [57].

The study found that pain or soreness in the upper limbs was significantly associated with casting technique—specifically, elliptical or sidearm casting was linked to a higher likelihood of pain. In contrast, overhead or two-handed casting (which involve more balanced and natural motion) were associated with a lower risk. These differences were statistically significant (χ^2^ = 7.44; *p* = 0.006). Pain was also associated with the use of weighted lines or additional tackle (e.g., split-shot, weighted flies), with a significant association (χ^2^ = 4.51; *p* = 0.034). Additionally, rod grip style influenced pain outcomes—100% of anglers who held the rod with a finger on top reported upper limb pain, compared to only 34% and 30% among those using thumb-on-top or “V”-style grips, respectively (χ^2^ = 6.17; *p* = 0.046).

No non-modifiable risk factors (e.g., age) were found to be associated with pain following fly fishing. Prior history of upper limb orthopedic injuries also did not significantly increase the likelihood of experiencing pain. There was no significant difference in isolated shoulder pain between overhead/two-handed casting and elliptical/sidearm casting styles (χ^2^ = 3.05; *p* = 0.081). Similarly, the type of fly fishing (freshwater vs. saltwater) did not influence the anatomical distribution of pain (χ^2^ = 0.554; *p* = 0.457) [57].

In the study *Upper Extremity Pain and Overuse Injuries in Fly-Fishing* by Kuhn and Kuhn, the prevalence of upper limb pain was 36.4%, most commonly involving the shoulder, wrist/hand, and elbow [57]. The pain most often resolved within a day or a week. In Berend’s earlier survey, 26%, 18%, and 23% of respondents reported wrist/hand, elbow, and shoulder pain, respectively. His sample was also male-dominated (93%) but on average nearly 15 years younger than that in the Kuhn & Kuhn study [66,74].

In a study by McCue et al., nearly half (49.8%) of professional fly-fishing instructors reported shoulder pain, 39% reported elbow pain, and 36% experienced wrist pain. Like previous findings, most of these individuals reported pain lasting only several hours (58%) or days (28%). However, their sample consisted of professional instructors who spent an average of 100 days per year casting, compared to 30 days in Kuhn & Kuhn’s cohort. McCue’s study did not report the median number of daily casts, whereas Kuhn & Kuhn found it to be approximately 200 per day [57,64,65,66].

#### 4.5.3. Lower Limb Pain

Leg pain, particularly in the knees, was reported by 23% of surveyed anglers. The prevalence of this issue varied depending on the type of fishing practiced—lowest among warmwater anglers (12%) and highest among saltwater anglers (31%). These differences are likely attributable to variations in technique and physical demands [74].

Warmwater fishing is often conducted while seated, which minimizes weight-bearing strain on the lower extremities. In contrast, fishing in rivers or marine environments typically requires anglers to wade or stand for extended periods on uneven or slippery surfaces. This prolonged static posture, combined with the instability of rocky or shifting terrain, can place considerable stress on the knees, ankles, and feet, potentially leading to pain, joint overload, or overuse injuries [74].

The repetitive loading involved in stepping, balancing, and adjusting stance while casting—especially in current or surf—can contribute to musculoskeletal discomfort in the lower limbs. In addition, prolonged cold exposure, often associated with river and coastal fishing, may exacerbate joint stiffness and reduce proprioceptive feedback, further increasing the risk of falls, slips, and strain-related injuries [74].

These observations highlight the importance of proper footwear, dynamic balance training, and rest periods for anglers who engage in physically demanding fishing environments. Further research is warranted to assess biomechanical load and injury risk specific to lower limb joints in various fishing contexts.

**Spinal Injuries and Disorders**

In a study of fly fishers operating in both freshwater and saltwater environments, the most commonly reported complaint was sciatica and lower back pain, stooping-related lumbar strain colloquially referred to as “Stooper’s back,” affecting 59% of respondents. The primary causes of back pain included prolonged standing, wading in fast-flowing rivers, uneven and slippery substrates (rocky, stony, or unstable terrain), and the prolonged use of heavy fishing vests [74].

The incidence of lower back pain among fly anglers appears comparable to that observed in other high-demand sports disciplines, such as tennis, where prevalence rates of up to 38% have been reported in professional players [85]. This suggests that the musculoskeletal demands associated with fly fishing—particularly repetitive trunk rotation, prolonged standing, and casting under variable environmental conditions—may place considerable strain on the lumbar spine.

Supporting this, a systematic review of back pain in sports reported prevalence rates of low back pain ranging from 1% to 94%, depending on the discipline, athlete age, and training load, with average values around 30% in adolescent athletes. For example, LBP is common among youth athletes engaged in asymmetric or impact-heavy sports such as baseball (3–15%), volleyball (30%), and judo (23.3%) [86]. These figures indicate that lumbar overuse is a relevant risk across both contact and non-contact sports, reinforcing the importance of recognizing and addressing spinal stress in fly anglers as part of broader musculoskeletal health strategies [85,87].

Interestingly, while the use of lumbar support belts did not reduce the overall incidence of back pain, it did lead to a reduction in the number of pain days per month among users. This indicates that while lumbar belts may not prevent the onset of pain, they may offer partial relief by limiting the severity or duration of symptoms [86].

The repetitive flexion, rotation, and prolonged axial loading associated with fishing—particularly when standing in cold water for hours or frequently bending to handle equipment—can contribute to mechanical strain, intervertebral disk pressure, and lumbar muscle fatigue. These factors, if unaddressed, may predispose anglers to chronic low back pain or aggravate pre-existing degenerative spinal conditions [74].

Preventive strategies, including strengthening of the core musculature, ergonomic load distribution (e.g., switching from traditional vests to lightweight chest packs), and awareness of posture during casting and landing, may help reduce the burden of spinal complaints in regular anglers.

### 4.6. Acute and Penetrating Injuries

Although fly fishing is often perceived as a recreational and low-risk activity, fishing in general carries the potential for acute injuries, some of which may require urgent medical attention. These injuries most commonly affect the upper limb, as shown in a large-scale epidemiological study by Joseph et al., which found that the incidence of upper extremity injuries related to fishing in the United States was 119.6 per 1 million person-years [88].

The most commonly injured site was the finger (63.3%), followed by the hand (20.3%). The leading mechanism of injury was foreign body penetration (70.4%), most often caused by fish hooks. The incidence of upper limb injuries was significantly higher in men (200 per 1 million person-years) compared to women (41 per 1 million person-years). A penetrating fish-fin spine injury, although often perceived as minor, can endanger critical neurovascular structures—as in this case of ulnar nerve paresthesia—and therefore requires prompt surgical exploration, removal of the foreign body, and appropriate prophylactic management. Such cases highlight that even seemingly trivial fishing-related wounds may carry significant risks and must not be underestimated. Given the widespread popularity of fishing, it is important that emergency physicians and hand surgeons are familiar with the appropriate evaluation and treatment strategies for such injuries [75,88,89,90].

Ocular trauma represents another category of severe acute injury in angling. According to a registry-based study from the United States, fishing accounted for nearly 20% of all sports-related eye injuries, with approximately 21% of cases resulting in visual acuity loss below 20/200. The most frequent mechanisms included penetration by hooks, lures, or sinkers, often requiring urgent ophthalmologic care and sometimes surgical intervention [91,92,93,94,95,96,97,98].

Recreational fishing is a popular activity among youth, with an estimated 14.8 million individuals under the age of 25 participating in the United States alone. However, this activity carries a notable risk of injury, particularly among adolescent males. An analysis of data from the National Electronic Injury Surveillance System (NEISS) covering the years 1997–2016 identified over 412,000 fishing-related injuries treated in emergency departments. The vast majority of injuries involved males (81.4%) and youth aged 11 to 18 years (53.7%). The leading cause of injury was contact with hooks and lures (79.7%), and the most frequently affected body parts were the upper extremities—especially the hands and arms (43.9%). These findings highlight the need for targeted injury prevention strategies, including educational initiatives aimed at older male adolescents, promotion of protective clothing, and potential design modifications to fishing equipment to reduce hook-related trauma [99].

From 2009 to 2014, 8220 patients with fishing-related injuries were identified in U.S. emergency databases, 85 of whom (1.03%) were linked to ice fishing. The proportion of injuries attributed to ice fishing rose from 0.52% in 2009 to 1.09% in 2014. The majority of these incidents occurred in January (38.8%), with all ice fishing injuries recorded between November and April.

Patients injured during ice fishing were on average older (39.4 ± 17.5 years) compared to those injured during traditional fishing (36.6 ± 25.1 years). Ice fishing injuries more frequently involved the trunk (*p* < 0.001), while upper limb injuries were more common in traditional fishing contexts (*p* < 0.001). Most injuries were classified as musculoskeletal trauma (45.9%) or minor trauma (36.5%), with frostbite-related injuries accounting for a smaller proportion (4.7%) [72].

Alcohol intoxication was reported twice as frequently in ice fishing cases (1.2%) compared to traditional fishing (0.6%). Although extremity injuries were less common in the ice fishing group, these injuries were generally more severe, with a greater likelihood of hospitalization (10.6% vs. 2.6%, *p* < 0.001). Notably, no deaths were recorded in the ice fishing group, compared to two fatalities among traditional anglers (*p* = 1.0) [72].

Among penetrating injuries associated with recreational and sport fishing, particular attention must be given to trauma involving the periocular region, including the eyelids and the globe itself. Although relatively uncommon, such injuries can result in serious complications and often require urgent surgical intervention. Reported cases range from superficial eyelid lacerations to deep penetration of the cornea, iris, and ciliary body, occasionally accompanied by secondary retinal detachment. Due to the structural density of some fishhooks, removal may necessitate advanced surgical approaches such as the “advance-and-cut” or the more recently described “clamp and retract” technique. Despite the severity of these injuries, favorable anatomical and functional outcomes, including full restoration of visual acuity, are achievable with timely and appropriate management. Therefore, any suspected fishhook-related ocular injury should be treated as a surgical emergency and referred immediately for ophthalmologic evaluation and intervention [93,100,101,102].

Although fishing is generally considered a low-risk recreational activity, it can be associated with rare but potentially fatal traumatic events. A remarkable case involved a 56-year-old man who died from severe blunt chest and abdominal trauma after being struck by a large Spanish mackerel (*Scomberomorus commerson*) that leapt into his recreational fishing boat. Autopsy findings revealed multiple critical injuries, including rib fractures, diaphragmatic rupture, mesenteric and intestinal contusions, pancreatic trauma, and splenic lacerations, resulting in hemothorax and hemoperitoneum. This incident occurred in the context of predator-induced fish escape behavior, with reports of shark-pursued mackerel jumping in the harbor on the same day. The case underscores the need to consider not only equipment-related and environmental risks but also unexpected interactions with large, fast-moving marine species when evaluating the safety of fishing activities [103].

Rock fishing is considered one of the highest-risk forms of recreational angling, accounting for a disproportionate number of drowning deaths and severe traumatic injuries along coastal rock platforms. Most fatalities occur when anglers are swept from rock ledges by unexpected waves, often in the absence of personal flotation devices or adequate footwear. Hospital data indicate that non-fatal rock fishing injuries commonly involve falls, blunt trauma, and submersion-related events, frequently requiring emergency intervention [104]. Drowning has also been identified as a critical acute risk in recreational fishing, particularly among anglers using waders, where altered buoyancy and loss of balance can contribute to hazardous submersion events [105]. Longitudinal survey data from New Zealand rock fishers showed substantial improvements in life jacket use following a regional safety campaign, although risky behaviors such as alcohol consumption and overreliance on local knowledge persisted, underscoring both progress and ongoing challenges in preventing drowning-related fatalities [106,107,108,109].

**Figure 2:**
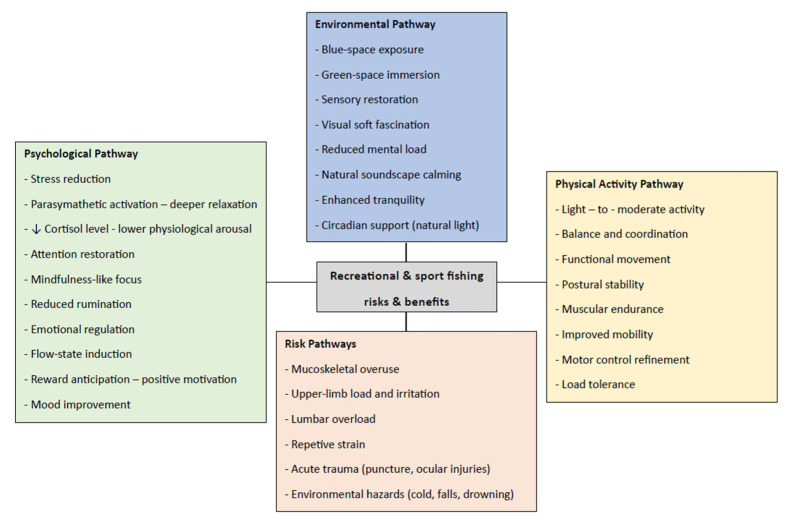
Integrated Psychological, Physical, and Environmental Pathways Associated with Recreational and Sport Fishing. The arrow represents a decrease in cortisol levels.

A conceptual model summarizing the integrated pathways identified in this narrative review is presented in Figure 2.

## 5. Final Considerations

This review provides a multidimensional synthesis of the health benefits and risks associated with recreational and sport fishing. At the same time, fishing is associated with distinct injury risks, including musculoskeletal overuse injuries, acute trauma (e.g., hook punctures, eye injuries), and rare but severe incidents such as drowning or hypothermia [58,75,100,110]. Moreover, the findings highlight fishing’s therapeutic potential and its underutilization in health promotion strategies. Programs such as Casting for Recovery and Project Healing Waters demonstrate that angling can serve as a tool for psychological rehabilitation and community reintegration. Fishing also offers opportunities to promote green exercise, which is linked to improved mental health outcomes and increased adherence to physical activity [17,19,20,23,25,27].

Understanding the health effects of angling requires situating the findings within established theoretical frameworks. The psychological benefits reported across studies are consistent with green-exercise and blue-space theories, which propose synergistic effects of physical activity and natural environments on stress reduction, autonomic regulation, and emotional restoration. These mechanisms align with the biopsychosocial model, in which outdoor activity influences physical, psychological, and social components simultaneously [18,51]. Psychologically, angling integrates nature immersion and focused attention in ways distinct from team sports or gym-based activities. These elements create a unique balance of therapeutic potential and injury risk not seen in other recreational disciplines [27,49]. Conversely, the musculoskeletal and overuse-injury patterns observed in anglers reflect well-described frameworks in occupational ergonomics and sports medicine, including the load–capacity model, repetitive-strain theory, and biomechanical concepts of mismatch between task demands and tissue capacity. Integrating these theoretical perspectives provides a clearer conceptual foundation for interpreting both the benefits and risks associated with recreational and sport fishing [56,80,81,82].

These contrasts illustrate that angling does not fully conform to traditional sports models, requiring a hybrid interpretive approach that integrates environmental psychology with occupational load frameworks.

To strengthen the practical relevance of the findings, we have expanded the manuscript with concrete recommendations. First, public health institutions may consider integrating fishing-based programs into local mental-health, rehabilitation, and social-inclusion initiatives, particularly for individuals experiencing chronic stress, long-term conditions, mood disorders, or for youth at risk. Second, safety strategies—including education on cold exposure, ergonomic casting techniques, and appropriate equipment selection—should be incorporated into national and regional angling guidelines. Third, partnerships between healthcare systems, schools, and angling organizations could support the implementation of structured programs for youth, veterans, and older adults, accompanied by instructor training, outcome monitoring, and long-term evaluation. Finally, national angling federations and associations could develop systems for monitoring injuries and participation patterns, enabling more precise prevention strategies and supporting data-driven policy development.

### 5.1. Future Research Directions

In addition to the overarching need for more systematic research, several concrete directions should be prioritized to strengthen the evidence base in this field. Future studies should employ large-scale prospective cohort designs and randomized controlled trials to establish causal relationships between angling participation and health outcomes. Research should extend to underrepresented angling disciplines (such as spinning, feeder, carp, marine, and ice fishing) and to populations that have been largely absent from existing studies, including women, youth, and individuals from non-OECD regions. Developing standardized outcome measures for physical, psychological, and social health effects is essential to improve comparability across studies. Further work is also required to clarify mechanisms underlying fishing-related injuries and to evaluate preventive strategies tailored to specific fishing techniques. Finally, angling-based interventions should be systematically assessed within structured mental health, rehabilitation, and community programs, with long-term monitoring and evaluation to guide policy development and practical implementation.

To advance the field, future studies should therefore:

- Conduct large-scale prospective cohort studies and randomized trials to establish causal relationships between fishing and health outcomes;
- Expand research to underrepresented disciplines and populations;
- Develop standardized outcome measures for assessing physical, psychological, and social benefits and risks;
- Investigate mechanisms of fishing-related injuries and develop effective prevention strategies adapted to different techniques;
- Explore fishing as part of integrated mental health, rehabilitation, and community-based programs.

### 5.2. Methodological Considerations and Potential Biases

This review has several methodological limitations that should be acknowledged. A substantial portion of the available literature focuses on fly fishing, which is practiced by only 2–3% of anglers worldwide, resulting in potential overrepresentation of this discipline in the evidence base. Other fishing techniques, such as spinning, float, feeder, carp, marine, and ice fishing, remain underexplored, with only isolated reports available, making it difficult to generalize findings to all forms of recreational and sport fishing.

Study design limitations further constrain interpretation. Most studies are observational, cross-sectional, or rely heavily on self-reported data about fishing activity, injuries, and health benefits, which introduces recall bias and prevents causal inference. Randomized controlled trials (RCTs) and prospective cohort studies are rare, and several injury-related studies are based on retrospective analyses or case reports, likely underestimating prevalence and lacking follow-up data.

Population and language bias also limit generalizability. Much of the literature originates from English-speaking, high-income countries, potentially overlooking data from regions where angling is culturally significant but underrepresented in research. Women, children, and anglers from non-OECD countries are notably absent from most studies.

Finally, there is substantial heterogeneity in how outcomes are measured and fishing disciplines are defined, further complicating cross-study comparisons. Future research should prioritize well-designed, prospective and interventional studies across diverse populations and fishing methods to provide a more comprehensive understanding of fishing’s multidimensional health benefits and risks.

A cross-study comparison further illustrates the heterogeneity of the available evidence across health domains. Therapeutic fly-fishing programs consistently demonstrated moderate reductions in PTSD symptom severity, with community-based trials reporting mean decreases of approximately 20–30% in standardized checklists [14,15,18,24]. Surveys also indicated that 25–35% of fly anglers experience musculoskeletal overuse complaints, particularly involving the shoulder, elbow, and wrist [57,83].

Evidence for ice fishing is limited to single-country case reports, documenting risks related to hypothermia, frostbite, fractures, and lacerations without providing pooled effect sizes [72].

General recreational angling shows moderate to strong evidence linking participation with reduced stress, lower depression scores, and improved well-being, with wellbeing differences typically ranging from +0.3 to +0.5 SD relative to non-anglers [14,18,19,23,56]. Large retrospective emergency-department datasets indicate that acute hook-related injuries—particularly to the skin and eye—occur at rates of approximately 18–22 cases per 100,000 ED visits [93,96,97,100,102,111].

Physical activity–related outcomes show moderate evidence, with most angling activities expending 3–4 METs.

Pediatric angling research remains sparse; national ED registries confirm that hook injuries account for approximately 0.02% of all visits among children, while early descriptive reports suggest possible benefits for attention in youth with ADHD, though no controlled trials exist [99].

Marine and sea fishing are the least represented domains, with only isolated case reports documenting fatal events such as projectile fish strikes or drowning [103,104,107,108,109].

Although many studies describe positive outcomes, fishing does not provide uniform benefits for all individuals. In some cases, effects may be modest, inconsistent, or comparable to other outdoor activities, and for some participants angling may simply substitute rather than enhance physical activity. Certain therapeutic initiatives also illustrate a gap between statistical findings and lived experience. For example, although quantitative evaluations of Casting for Recovery have not consistently demonstrated strong effect sizes, thousands of breast cancer survivors worldwide report a deep sense of comfort, belonging, and emotional support within this community. These nuances underscore the need to interpret the evidence with caution and to recognize that meaningful benefits may extend beyond measurable clinical outcomes [16,35,36].

## 6. Conclusions

The available evidence indicates that—when practiced safely and with recognition of its physical and environmental demands—recreational and sport fishing can be conceptualized as a health-promoting outdoor activity with meaningful therapeutic potential. More balanced, methodologically rigorous research across fishing disciplines and populations will be essential to further clarify its role in public health, rehabilitation, and preventive medicine.

This review addressed the four objectives proposed at the outset. First, it synthesized current evidence on musculoskeletal, psychological, and environmental factors associated with recreational and sport fishing. Second, it identified key physical, mental, and social benefits across angling disciplines. Third, it characterized acute and chronic injury risks, including overuse syndromes and environmental hazards. Finally, it highlighted major gaps in the literature—such as limited evidence outside fly fishing, scarcity of prospective studies, and insufficient data from underrepresented populations—providing a roadmap for future research in public health, rehabilitation, and preventive medicine.

## Data Availability

Not applicable. No datasets were generated or analyzed during the current study.
